# A review on gut microbiota: a central factor in the pathophysiology of obesity

**DOI:** 10.1186/s12944-021-01491-z

**Published:** 2021-07-07

**Authors:** A. L. Cunningham, J. W. Stephens, D. A. Harris

**Affiliations:** 1 Department of Surgery, Swansea Bay University Health Board, Swansea, SA2 8QA UK; 2 Swansea University Medical School, Swansea University, Swansea, SA2 8QA UK

**Keywords:** Gut microbiota, Obesity, Metabolic syndrome, Faecal microbiota transplant

## Abstract

Obesity and its complications constitute a substantial burden. Considerable published research describes the novel relationships between obesity and gut microbiota communities. It is becoming evident that microbiota behave in a pivotal role in their ability to influence homeostatic mechanisms either to the benefit or detriment of host health, the extent of which is not fully understood. A greater understanding of the contribution of gut microbiota towards host pathophysiology is revealing new therapeutic avenues to tackle the global obesity epidemic. This review focuses on causal relationships and associations with obesity, proposed central mechanisms encouraging the development of obesity and promising prospective methods for microbiota manipulation.

## Background

The worldwide prevalence of obesity has approximately tripled since 1975 with a current estimate of 1.9 billion adults being classed as overweight (body mass index, BMI ≥ 25 kg/m^2^). This currently outnumbers those with malnutrition [1, 2]. Obesity is defined as the ‘abnormal or excessive fat accumulation that may impair health’ and is measured using the BMI [3]. Factors contributing towards the obesity epidemic include an increased accessibility to energy-dense foods, an increase in sedentary activity and the possible involvement of the gut microbiota on host metabolism.

Although the fundamental cause of obesity is an energy imbalance between the calories consumed and the calories expended [1], body weight is not influenced by the calorific ingestion*,* but rather by the calories that are absorbed [4]. When adipose tissue exceeds its buffering capacity to store excess triglycerides, a resulting overflow of lipids into the systemic circulation occurs [5]. This lipid overspill to non-adipose tissues such as the liver, skeletal muscle and pancreas culminates in ectopic fat storage and the subsequent development of insulin resistance. Secondly, inflammation in adipose tissue increases, triggering the production and secretion of pro-inflammatory cytokines and adipokines, which contribute to the development of peripheral insulin resistance and altered glucose homeostasis [5].

### Gut microbiota

In the early 1900s, Élie Metchnikoff, a Russian-born zoologist and microbiologist first postulated the theory that gut microbiota behave as central modulators influencing host homeostasis and metabolism [6]. He believed that disruption of host homeostasis by particular bacteria increased the possibility of a disease state resulting in systemic toxicity from bacterial by-products [6]. An adult human is colonised by approximately 100 trillion microbes, most of which are predominantly found in the gastrointestinal tract (GIT), the largest population residing in the colon [7].

Scientists are gaining a greater understanding of the ‘normal’ bacterial communities and physiology of present gut microbiota through population research such as the Human Microbiome Project [8].

Taxonomy is the study of classifying microbiota and provides a rigid structure for the arrangement of particular microbiota into groups on the basis of mutual similarity or evolutionary relatedness. In bacterial taxonomy the most commonly used ranks (levels) in ascending order are species, genera, families, orders, classes, phyla and domain [9].

**Fig. 1:**
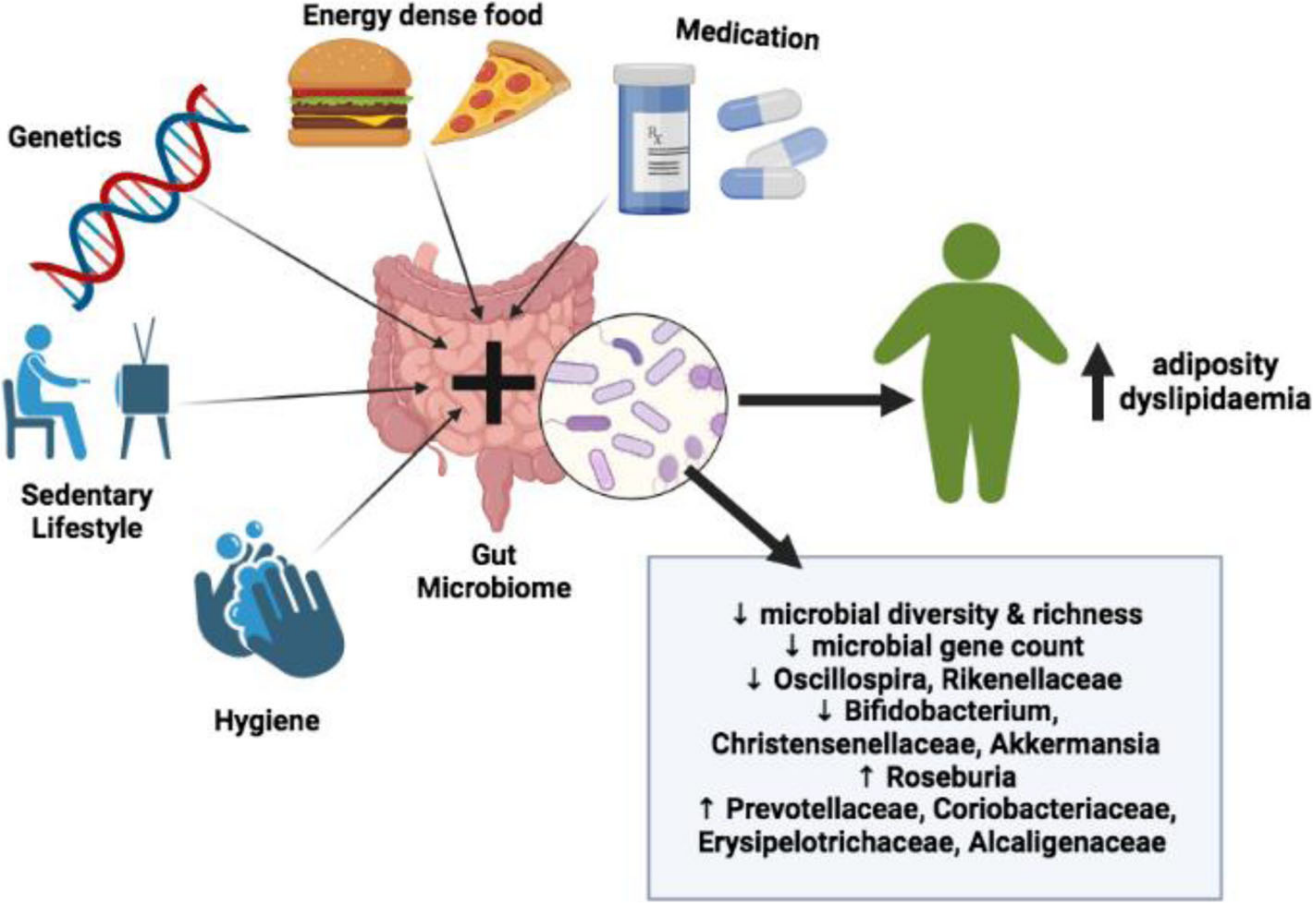
Contributions towards obesity development including gut microbiota findings

The most abundant faecal bacterial groups of both lean and obese subjects are the phyla Firmicutes and Bacteroidetes [10, 11]. Approximately 90% of all phylotypes of gut bacteria belong to either the gram-positive Firmicutes (64%) or the gram-negative Bacteroidetes (23%) [8, 12]. Other important phyla are Proteobacteria, Actinobacteria, Verrucomicrobia and Fusobacteria [8, 13–15]. The host genome is pivotal in controlling the composition of gut microbiota, however many external factors such as diet, illness, lifestyle, hygiene and the use of medications can contribute to changes in bacterial communities [16–18]. Growing evidence illustrates that dietary modification may be extremely influential in accounting for gut microbiota variations [17, 19, 20] (summarised in Fig. 1).

### Microbiota population differences in obesity

Maintaining the heterogeneity and stability within the gut microbiota community is essential for promoting host health. Alterations in diversity and microbiota community structure may affect host metabolism resulting in obesity. Subjects with obesity have consistently demonstrated a reduction in diversity and richness in microbial populations which can be reversed using weight loss interventions (diet low in fat and animal products, rich in fruit and vegetables) [21, 22]. Microbial diversity has been linked to the metabolic function of gut microbiota and low bacterial richness has been suggested to be a risk factor for obesity and low-grade inflammation [23, 24].

Obesity-related host microbiome display enrichment in particular gene categories involved in carbohydrate and lipid metabolism, and enzymes involved in glucose and insulin signaling pathways are down-regulated [11, 21, 25]. Le Chatelier et al., analysed gene counts of a large cohort of obese and healthy subjects. Subjects identified with a low gene count (LGC) showed traits typical of an ‘obese phenotype’ associated with greater overall adiposity, insulin resistance and dyslipidaemia [26]. LGC subjects also had increased levels of serum leptin, triglycerides and free-fatty acids, high density lipoprotein-cholesterol, decreased serum adiponectin and an elevated inflammatory phenotype [26, 27]. Dietary restriction among overweight or obese patients is less efficient in LGC than in high gene count individuals when targeting the improvement of insulin sensitivity and lowering of lipid and inflammatory biomarkers [26, 28].

The inconsistency across both animal and human studies regarding the Firmicutes/Bacteroidetes phyla ratio in obesity diminishes the significance of this particular finding. This review will therefore focus on more in-depth microbiota relationship findings below phylum level as summarised in Table 1.

**Table 1:** Gut microbiota differences in obese human and rodent cohorts, preoperative bariatric dietary cohort, post-bariatric surgery human and rodent cohorts, post-allogenic FMT cohort

Cohort	Microbiota Findings
Obese cohort [10, 11, 22, 29–32]	↓ *Rikenellaceae* and *Christensenellaceae* ↓ *Bifidobacterium*, *Oscillospira* and *Akkermansia*
Obese cohort [33]	↓ *Alistipes finegoldii* and *Alistipes senegalensis*
Obese cohort [34]	*Alistipes* = marker of persistent weight loss success
Obese cohort [11, 35]	↓ Faecali prausnitzii
Obese cohort [21, 32, 36]	Weight loss ↑ *Faecali prausnitzii*, *Akkermansia* and *Christensenellaceae*
Obese cohort [37]	↑ Prevotellaceae, Coriobacteriaceae, Erysipelotrichaceae, and Alcaligenaceae
Elevated BMI [38, 39]	↑ *Roseburia*
Obese cohort [40]	↑ *Eubacterium dolichum*
Obese cohort [37]	↑ H_2_-producing bacteria; (*Prevotellaceae*, certain groups within the Firmicutes and Archaea)
Obese cohort [11, 26]	↑ gram-negative microbes ↑ Fusobacterium, Escherichia-Shigella, Pseudomonas and Campylobacter
Obese children and overweight women [30, 41]	↑ *Staphylococcus aureus*
Obese cohort on preoperative diet [42]	↓ *Streptococcaceae* and *Ruminococcaceae* ↑ *Rikenellaceae* and *Bifidobacteriaceae*
Post-bariatric surgery [37, 42, 43]	↑ Gammaproteobacteria (*Enterobacteriaceae)* ↓ Firmicutes (*Clostridium difficile*, *Clostridium hiranonis*, and *Gemella sanguinis*) ↓ methanogens
Post-bariatric surgery [43, 44]	↑ *Escherichia*, *Klebsiella*, and *Pseudomonas*
Six months post-bariatric surgery [42]	↑ *Streptococcaceae* and *Veillonellaceae* ↓ *Bifidobacteriaceae*
Rodent model – post bariatric surgery [45]	↑ Bacteroidetes, Verrucomicrobia, and Proteobacteria ↑ *Alistipes*, *Akkermansia*, and *Escherichia*
Rodent model – post bariatric surgery [46]	↑ Proteobacteria (*Enterobacter hormaechei)* ↓ Firmicutes and Bacteroidetes
Allogenic FMT recipients [47]	↑ *Roseburia intestinalis*

A specific microbial signature associated with a diagnosis of obesity has still not been identified. The most common gut microbiota composition finding is a reduction in the butyrate-producing microbes together with an increase in opportunistic pathogens [29]. Consistent microbiota findings have displayed reductions in the abundances of the families *Rikenellaceae* and *Christensenellaceae* as well as a decrease in the abundance of the genera *Bifidobacterium*, *Oscillospira* and *Akkermansia* [10, 11, 22, 29–32].

Many of these depleted microbiota provide beneficial attributes to the host. *Bifidobacterium* is associated with elevated short chain fatty acids (SCFAs), decreased luminal lipopolysaccharide (LPS) and improved intestinal barrier function [31, 48]. The importance of these mechanisms are discussed in individual sections later in the review. Both *Christensenellaceae* and *Akkermansia* correlate with lower visceral fat mass, a type of fat that is considered to be an adverse cardiometabolic risk factor when in excess [24, 49]. *Akkermansia* is a mucin-degrading microbe inhabiting the outer mucus layer of the intestinal barrier and is associated with a healthier metabolic status in obese humans [24]. It has been reported that having increased amounts of *Akkermansia* in the gut prior to embarking on a weight loss programme leads to greater improvements in glucose homoeostasis, blood lipids and body composition [24].

In addition to the above findings, two species within the *Rikenellaceae* family have been identified that correlate negatively with BMI: *Alistipes finegoldii* and *Alistipes senegalensis* [33]. This finding has been replicated in a German weight-loss intervention study who enrolled female participants on a very low calorie diet and found that the genus *Alistipes* was a marker of persistent weight-loss success [34]. The species *Faecali prausnitzii* is also significantly reduced in obesity, particularly in those patients with diabetes [11, 35]. Weight loss in obese adults has been shown to have the reverse effects on microbiota composition and enhances the relative abundances of *Faecali prausnitzii*, *Akkermansia* and *Christensenellaceae* [22, 32, 36].. *Faecali prausnitzii* is an important butyrate-producing microbe and is understood to provide host protection against bacterial translocation [50].

Specific gut microbiota have also been reported to increase in obesity: - the families *Prevotellaceae*, *Coriobacteriaceae*, *Erysipelotrichaceae* and *Alcaligenaceae* [37]. Secondly, increased abundance of the genus *Roseburia* is consistently reported in subjects with elevated BMI [38, 39]. *Roseburia* has the ability to hydrolyse and ferment polysaccharides into SCFA, thereby increasing energy harvest from the diet [51]. Lastly, the species *Eubacterium dolichum* has been positively associated with visceral fat mass as a surrogate marker of obesity [40].

The generation of hydrogen (H_2_) in the GIT provides an inhibitory effect on gut microbiota resulting in reduced fermentation [52]. H_2_-producing bacteria; originating mainly from the *Prevotellaceae* family, certain groups within the Firmicutes and H_2_-oxidizing methanogenic Archaea, have been found to be in significantly greater numbers in microbiota communities of obese subjects [37]. Archaea are not bacteria but comprise a separate taxonomic kingdom and are organisms consisting of a single cell without a nucleus [53]. Methanogens readily absorb H_2_ allowing for continued carbohydrate fermentation by H_2_-producing bacterial groups resulting in greater SCFA availability and an increased availability of calories [37, 54].

As discussed later in this review, gram-negative bacteria provide a ready source of LPS, increasing the likelihood of systemic host inflammation. Gram-negative bacteria including the genera *Fusobacterium*, *Escherichia-Shigella*, *Pseudomonas* and *Campylobacter* are all prevalent in obesity [11, 26]. The family *Prevotellaceae*, which provides a source of bacterial LPS is significantly enriched in obese subjects [37]. Lastly, the species *Staphylococcus aureus,* a well-known opportunistic pathogen, was found to be in reduced quantities in children who maintain normal weight compared with children that are overweight several years later [41]. This finding was also replicated in a cohort of overweight women [30].

It is clearly demonstrated that a reduction in gut microbiome diversity occurs in obese subjects, but there are still many unanswered questions on the precise microbial population of an obese gut microbiome. Whether it is more important to focus on microbiota composition at phyla or deeper levels such as genus and species remains open to debate and if the absence, depletion or presence of particular microbiota contributes to the development of obesity.

### Microbiota contribute to the development of obesity

The exact mechanisms by which ‘obese microbiota’ influence the development of obesity is still unfolding. Animal models have been widely utilised for the in-depth analysis of the microbe-host relationship and allow for the investigative impact of microbiota interventions in the pathogenesis of obesity. In this section we explore the basic involvement of the gut microbiome in mouse models before focusing on the potential mechanisms.

Germ free (GF)-mice display attributes indicating the possibility of resistance to developing obesity induced by consuming a high-calorific diet strongly suggesting a possible causal role for gut microbiota [55]. Allowing both GF and conventional mice (C57BL/6) access to unlimited chow, it was observed that GF-mice exhibited considerably less (42%) total body fat than conventional mice, despite ingesting a daily consumption of 29% more chow [56]. Lupp et al. [57], also analysed GF-mice and observed that this particular cohort weighed significantly less and eliminated twice as many calories in their stool compared with their conventional counterparts. Together, these findings demonstrate that GF-mice harvest less energy from their diets suggesting that the presence of the gut microbiome increases energy harvest.

To better understand the gut microbiome involvement, GF-mice (C57BL/6), were colonised with gut microbiota extracted from conventionally raised obese mice. Within fourteen days, the GF-mice had subsequently increased their total body fat content by 60% with associated insulin resistance, despite ingesting reduced amounts of chow [56]. Several possible mechanisms were proposed: - increased microbial fermentation of dietary polysaccharides that could not previously be digested; subsequent increase in intestinal absorption of both monosaccharides and SCFAs; and microbial regulation of host genes that promote deposition of lipids in adipocytes [56].

Turnbaugh et al. [58], performed microbiota transplantation into GF-mice (lean) with microbiota extracted from both obese and lean mice. All mice were allocated equal amounts of chow. Fourteen days post-procedure, the cohort who had received ‘obese’ microbiota had increased their total body weight compared to those who had received ‘lean’ microbiota who remained lean [58]. The investigators also examined the ‘obese’ rodent’s genome and discovered significant enrichment of gene tags (GLB1: β-galactosidase, melA: α-galactosidase, GAA: α-glucosidase, PFKL: 6-phosphofructokinase) encoding for enzymes involved in the degradation of dietary polysaccharides that would be otherwise indigestible [58].

Ridaura et al. [59], were the first group to perform human faecal microbiota transplantation (FMT) on a cohort of GF-rodents. Gut microbiota were extracted from adult human female twin pairs discordant for obesity. Initially, all GF-rodents were considered to be of normal weight, allocated low-fat chow and co-housed. Rodents who received ‘obese’ FMT exhibited growth in their total body and fat mass while those who received ‘lean’ FMT remained lean. Stool sequencing indicated successful integration of the human donor microbiota, including the transfer of functions associated with the respective lean or obese microbial communities [59].

### Homeostatic mechanisms influenced by gut microbiota in the development of obesity

**Fig. 2:**
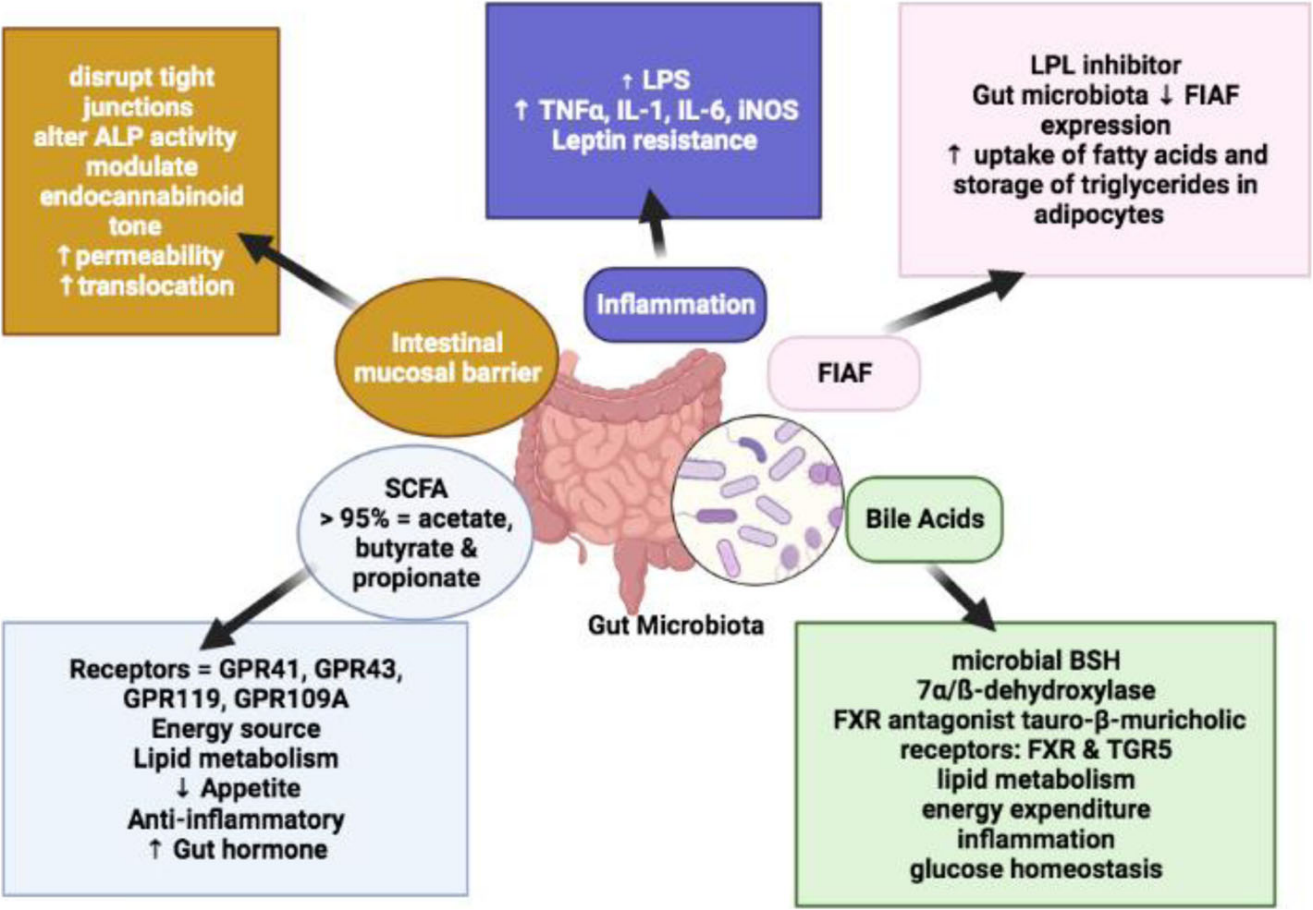
Host metabolic pathways influenced by gut microbiota and metabolites including SCFAs, Fiaf inhibition, bile acid metabolism, intestinal mucosal barrier and host inflammatory pathway

Gut microbiota have the capability to impact host physiology both to its benefit and detriment either directly or via microbial metabolites [60, 61]. Almost 10% of all circulating metabolites in an adult human are derived from microbiota and participate in metabolic pathways [51]. We discuss the most important mechanisms involving the contribution of gut microbiota leading to the development of host obesity (summarised in Fig. 2).

### Short chain free fatty acids

The production of SCFAs by microbial fermentation has been linked to reductions in body weight and adiposity [62]. SCFAs are small organic monocarboxylic acids and constitute the major microbial metabolites produced during anaerobic carbohydrate fermentation in the gut [63]. SCFAs consist of one to six carbons of which acetate (C2), propionate (C3) and butyrate (C4) are the most abundant (≥95%) [64]. GF-mice are devoid of SCFAs, highlighting the central role of microbiota in the production of SCFAs [65, 66]. SCFAs target host metabolic signaling pathways through coupling action with selected G-protein-coupled receptors (GPR41, GPR43, GPR119, GPR109A), which are abundant in adipocytes, gut immune cells and epithelial cells [67–70].

These receptors are not stimulated equally by all SCFAs. Propionate primarily activates GPR41, butyrate activates GPR109A, whereas GPR43 and GPR119 can be activated by acetate, butyrate and propionate at similar rates [70, 71]. The order *Clostridiales* provides the largest microbiota population towards the production of SCFAs including the genera *Anaerostipes, Clostridium, Coprococcus, Dorea, Eubacterium, Faecalibacterium, Roseburia, Ruminococcus, Peptococcus*, and *Peptostreptococcus* [72].

The activation of receptors GPR41 and GPR43 induces the secretion of peptide tyrosine-tyrosine (PYY), reducing host appetite by directly stimulating the central nervous system [73, 74]. GPR41 coupling has the ability to initiate the expression of leptin from adipocytes [75, 76] and was first discovered using GPR41 deficient mice that displayed substantially lower leptin levels than corresponding wild-type mice [77]. Leptin acts on the hypothalamus, reducing food intake by inhibiting the release of neuropeptide Y (NPY) and promotes an increase in host metabolic rate consequently enhancing energy expenditure.

GPR43 receptor coupling with acetate directly reduces lipolysis in adipocytes, decreasing plasma-free fatty acids suggesting a possible therapeutic role for receptor GPR43 in lipid metabolism regulation [78]. Studying wild-type rodents, the receptor GPR43 was found to be mainly expressed in immune and white adipose tissue (WAT). Observing the activation of GPR43 in WAT found that insulin-induced protein kinase B (AKT)-activation was significantly reduced, resulting in less fat accumulation. In conclusion, GPR43-deficient rodents are phenotypically obese in contrast to rodents with a specific overexpression of GPR43 in WAT, who remain lean even when subjected to a high calorific diet [38].

SCFA coupling with receptors GPR43 and GPR109A provides anti-inflammatory protection for the host. GPR43 coupling promotes the production of antimicrobial peptides RegIIIγ and β-defensin and immunity-related cytokines such as interleukin (IL)-1, IL-6, IL-12 and IL-18 [39, 79]. GPR109A activation suppresses colonic inflammation and carcinogenesis by promoting anti-inflammatory properties in colonic macrophages and dendritic cells, which induce the differentiation of regulatory and IL-10-producing T cells [80].

Acetate, the most abundant SCFA is produced by enteric bacteria from the genera Lactobacillus, *Bifidobacterium, Akkermansia, Bacteroides, Prevotella, Ruminococcus* and *Streptococcus* [81]. Acetate is readily absorbed, transported to the liver to be used as an energy source and also utilised as a substrate for the synthesis of cholesterol and long-chain fatty acids [82]. The presence of acetyl-coenzyme A synthetase in adipose tissue allows for the use of acetate in lipogenesis on entering the systemic circulation [64]. Large quantities of systemic acetate may enhance host production of the ‘hunger hormone’, ghrelin, which is released from enteroendocrine cells in the GIT worsening hunger, reducing metabolic rate and increasing gastric motility and gastric acid production [83].

Butyrate has particular importance in host homeostasis and may contribute to the regulation of body weight. The genera *Anaerostipes, Clostridium, Coprococcus, Dorea, Eubacterium, Faecalibacterium, Roseburia and Ruminocococcus* all produce butyrate*.* The most abundant producers appear to be the species *Faecalibacterium prausnitzii, Eubacterium rectale* and *Roseburia intestinalis* [84, 85]. *Eubacterium* and *Anaerostipes* have the ability to interact with *Bifidobacterium* to enhance their butyrate production capacity [86].

Butyrate promotes energy expenditure, possibly reducing obesity through the enhancement of mitochondrial activity (activates AMP-activated protein kinase, increasing adenosine triphosphate (ATP) consumption and the induction of peroxisome proliferator-activated receptor gamma coactivator one (PGC-1) activity), in association with the up-regulation of the expression of genes involved in lipolysis and fatty acid oxidation [87, 88]. Butyrate supplementation in rodents maintained on a high-calorific diet has been observed to prevent the development of dietary obesity and insulin resistance [89]. At cellular level, butyrate was noted to increase mitochondrial respiration [89]. Butyrate may reduce energy intake by invoking a host anorectic response by increasing the plasma levels of glucagon-like peptide 1 (GLP-1), glucose dependent insulinotropic polypeptide (GIP) and PYY [87, 88].

Propionate is mainly produced by the genera *Phascolarctobacterium*, *Bacteroides*, *Dialister*, *Megasphaera*, *Veillonella*, *Coprococcus, Roseburia, Ruminococcus* and *Salmonella* [81]. Propionate stimulates the release of the anorectic gut hormones PYY and GLP-1 and has the ability to stimulate a gut–brain circuit through the action of receptor GPR41 thus leading to the induction of intestinal gluconeogenesis (IGN) gene expression. Up-regulation of IGN by propionate reduces body weight gain and adiposity, independent of food intake [90]. Providing propionate supplementation, results in a reduction in weight, abdominal adipose tissue and hepatic fat; an improvement in insulin sensitivity; increased satiety and reduced appetite [91, 92].

### Reduced activity of fasting-induced adipose factor

Fasting-induced adipose factor (Fiaf) is a circulating lipoprotein lipase (LPL) inhibitor produced by the intestine, liver and adipose tissue [93]. Gut microbiota efficiently suppress Fiaf expression in the ileum, enhancing the activity of LPL and increasing cellular uptake of fatty acids and storage of triglycerides in adipocytes [56]. LPL is the key enzyme that acts on the endothelial surface of extra-hepatic capillaries, releasing large amounts of fatty acids from lipoproteins for the uptake of tissues for production or storage of energy [94]. Bäckhed et al., demonstrated that hepatic lipogenesis appears to be induced in conventionalised GF-mice (CONV-D). The importance of Fiaf in this regulatory pathway was clarified by comparing GF- Fiaf knockout (Fiaf−/−) rodents to their wild-type littermates. In the absence of Fiaf, rodents gained substantially more weight than their littermates due to enhanced LPL activity. It is therefore possible that a reduction in Fiaf activity was responsible for the increased adiposity in CONV-D mice [56].

### Bile acids

Bile acids (BA) are cholesterol-derived metabolites produced in hepatocytes, which are conjugated to glycine or taurine and 95% reach the entero-hepatic circulation following reabsorption in the terminal ileum [95]. The primary BAs chenodeoxycholic acid (CDCA) and cholic acid (CA) are essential for lipid/vitamin digestion and absorption. Small quantities of primary BAs reach the colon where gut microbiota have the capability to convert them into secondary BAs through the processes of deconjugation, dehydroxylation, and reconjugation. Examples are deoxycholic acid (DCA), ursodeoxycholic (UDCA) and lithocholic acid (LCA) [96]. In the colon, conjugated CA and CDCA are deconjugated and then dehydroxylated at the 7α-position for the formation of the secondary bile acids DCA and LCA, respectively [97].

BA deconjugation is catalysed through the enzymatic activity of bile salt hydrolases (BSH) found within gut microbiota particularly from the genera *Lactobacillus*, *Bifidobacterium*, *Enterobacter*, *Bacteroides* and *Clostridium* [98, 99]. Microbial BSH is an enzyme which enhances BA intestinal reabsorption, promotes intestinal colonisation and can provide a nutritional source of sulfur, nitrogen and carbon [96]. Unlike BSH activity, only a small number of genera such as *Clostridium* and *Eubacterium* possess the enzyme 7α/ß-dehydroxylase which participates in the conversion of primary to secondary BAs [96]. Gut microbiota also possess the capability to influence BA synthesis through the metabolism of the naturally occurring FXR antagonist tauro-β-muricholic acid [100, 101].

Disruption to the microbiota population strongly affects BA metabolism leading to a failure in the conversion of primary BAs, resulting in their accumulation [102, 103]. Both primary and secondary BAs exert their biological effects by activating nuclear and plasma membrane receptors (nuclear farnesoid X receptor (FXR) or the G protein-coupled receptor (TGR5)) which control the synthesis and metabolism of BAs. Primary BAs bind to the FXR receptor whereas secondary BAs bind to the TGR5 receptor [99]. Stimulation of these receptors initiates signaling cascades and activates gene expression involved in the regulation of glucose homeostasis, lipid metabolism, energy expenditure and inflammation [96, 104].

Receptor TGR5 is expressed in brown adipocytes, macrophages, hepatic Kupffer cells, gallbladder epithelium and the colon [105]. TGR5 signaling enhances energy expenditure in adipose tissue through the action of cyclic adenosine monophosphate (cAMP) increasing the induction of type 2 deiodinase (DIO2), which converts and activates thyroid hormone T_4_ to T_3_ to stimulate energy metabolism in mitochondria. In the colon, the action of cAMP stimulates the release of GLP-1 in L cells. TGR5 signaling also has the ability to suppress inflammation in macrophages, the intestine and hepatocytes by inhibiting nuclear factor kappa-light-chain-enhancer of activated B cells (NF-κB) translocation, antagonising tumour necrosis factor alpha (TNFα) and NF-κB-dependent induction of pro-inflammatory cytokines [105].

Stimulation of the FXR receptor induces gene expression involved in lipogenesis and de novo cholesterol synthesis including apolipoprotein C-II, apolipoprotein E, peroxisome proliferator-activated receptor alpha (PPARα) and syndecan-1 [105]. Secondly, FXR stimulation triggers a reduction in very low density lipoprotein (VLDL) production by inhibiting the mitochondria triglyceride transport protein which is necessary for the assembly of VLDL particles [102]. Thirdly, FXR receptor signaling improves reverse cholesterol transport by enhancing the activity of the phospholipid transport protein which transports cholesterol from peripheral tissues to the liver for the conversion into BAs, thus preventing the accumulation in macrophages. Lastly, FXR stimulation induces fibroblast growth factor 19 (FGF-19) synthesis which inhibits BA synthesis, increases host metabolic rate, induces the hepatic leptin receptor and increases fatty acid oxidation [105]. Introducing the FXR agonist obeticholic acid was found to improve obesity-related disorders in an animal model [106].

The participation of the gut microbiota in the metabolism of BAs strengthen its role as a central regulator of lipid metabolism contributing to the progression of host obesity. Further research is required to clarify the central role of the gut microbiome in regulating BAs as an important mechanism towards the pathogenesis of obesity.

### Intestinal mucosal barrier

The mucosal lining of the GIT acts as a preventative barrier, reducing undesirable interactions between the gut epithelium, viruses, toxins and pathogenic bacteria [107]. It is well established that obesity is associated with mucosal barrier dysfunction with increased permeability and greater levels of systemic LPS in obese rodents [48]. Disruption of the mucosal lining allows for the translocation of toxins, resulting in metabolic endotoxaemia and subsequent low-grade inflammation, autoimmunity and oxidative stress [108, 109].

Gut microbiota take part in colonisation resistance which reinforces the mucosal barrier against colonisation by pathogens and provide continuous stimulation of pathogen recognition receptors to increase the production of mucins and antimicrobial peptides [110]. Mucosal adherent microbiota, such as the species *Akkermansia muciniphila*, are important for homeostatic epithelial cell stimulation [24]. Other microbiota have also been consistently reported to benefit gut barrier function. Both the genus *Roseburia* and the species *Faecali prausnitzii* are important butyrate-producing microbes which are well understood in their ability to provide protection against bacterial translocation [50, 51]. Lastly, an increased abundance of the genus *Bifidobacterium* has been associated with reduced gut leakiness and a reduction in serum LPS [31, 48].

GIT mucosal function is maintained via several mechanisms including appropriate localisation and distribution of tight junction proteins, normal endocannabinoid system tone, and LPS detoxification by intestinal alkaline phosphatase. The presence of SCFAs enhances gut barrier integrity [109, 111]. Gut microbiota have the ability to disrupt tight junction proteins, alter alkaline phosphatase activity and selectively modulate colonic expression of the cannabinoid receptor 1 (CB1), strongly impacting permeability through effects on zonula occludens-1 and occludin [112, 113]. Brun et al., analysed cross-sections of intestine obtained from obese rodents which demonstrated a reduction in the tight junction protein occludin and an irregular distribution of zonula occludens-1 [114].

### Inflammatory response

Obesity is associated with a state of chronic low-grade inflammation with abnormal expression and production of multiple inflammatory mediators [115–117]. Inflammation in metabolic disease was first described by Hotamisligil et al., who demonstrated the ability of adipocytes to express the cytokine TNFα and that TNFα expression in adipocytes of obese animals is intensified [118]. Gut microbiota exacerbate inflammation through the activity of LPS, an essential component of the cell walls of Gram-negative bacteria [119–121]. The genera *Fusobacterium*, *Escherichia-Shigella*, *Pseudomonas* and *Campylobacter* are all prevalent in obesity [11, 26]. LPS from members of the families *Enterobacteriaceae* and *Desulfovibrionaceae* exhibit an endotoxin activity that is 1000-fold greater than LPS from *Bacteroideaceae* [122].

Dietary fat, which is incorporated into triglycerides combines to form larger chylomicrons for systemic transportation, has a high affinity for LPS. Intestinal absorption of dietary fat therefore facilitates the direct movement of LPS into the systemic circulation [119]. Once in the circulation, LPS is recognised and triggers both the innate and local immune response and the subsequent release of pro-inflammatory molecules TNFα, IL-1, IL-6, and inducible nitric oxide synthase (iNOS) [16]. LPS is also believed to play a role in host development of leptin resistance [123], causing hyperphagia and weight gain, further increasing fat intake, raising LPS and leading to further inflammation [124].

Obese rodents exhibit significantly greater levels of plasma LPS than their lean counterparts and also display low-grade systemic inflammation [125]. Subcutaneous injection of the species *Escherichia coli* LPS into wild-type rodents maintained on standard chow produced the development of inflammation, obesity, fasted glycaemia and insulinaemia. Importantly, in cluster of differentiation 14 (CD14)-knockout mice, in which LPS cannot be recognised by the innate immune system, there was a reduction or even a complete lack of development of most features of metabolic diseases induced by high calorific chow or a LPS infusion [125].

### Microbiota population differences after bariatric surgery

Bariatric surgery is currently the only available treatment for morbid obesity that consistently achieves and sustains substantial weight loss [126]. The Roux-en-Y gastric bypass (RYGB), is the most commonly performed bariatric operation and involves the creation of a small gastric pouch from the fundus of the stomach. The distal stomach and proximal small intestine are bypassed by anastomosing the distal end of the mid-jejunum to the proximal gastric pouch (creating the Roux limb), and then reattaching the biliary and pancreatic limb at a specified distance along the Roux limb [37, 44]. This procedure alters acid exposure to the gastric remnant and proximal small bowel, restricting the quantity and type of food that can be ingested comfortably and allows a degree of nutrient malabsorption by reducing the length of the small bowel. The resulting rise in pH, increase in oxygen, downstream delivery of bile acids and consequent alteration in food ingestion may contribute to the changes seen in the gut microbiota population [37, 42, 44].

Gut microbiota changes after bariatric surgery have been reported in both rodent models and humans. No significant differences in diversity or relative abundance have been demonstrated between the differing bariatric procedures, a RYBG or sleeve gastrectomy [42]. In preparation for surgery, patients are instructed to follow a preoperative diet, which significantly adjusts the gut microbiota population. A reduction in the abundance of the families *Streptococcaceae* and *Ruminococcaceae* was noticed alongside a significant increase in *Rikenellaceae* and *Bifidobacteriaceae* [42]. After surgery, large increases in Gammaproteobacteria (96.2% of which were members of the family *Enterobacteriaceae*), a proportional decrease in Firmicutes (the species *Clostridium difficile*, *Clostridium hiranonis*, and *Gemella sanguinis*), and a loss of methanogens was reported [37, 42, 43]. Several facultative anaerobes in the Proteobacteria (the genera *Escherichia*, *Klebsiella*, and *Pseudomonas*) were also discovered at enhanced levels [43, 44]. An increase in the abundance of the families *Streptococcaceae* and *Veillonellaceae* and a decline in *Bifidobacteriaceae* that persisted for at least six months after surgery have also been observed [42].

The Bacteroides/Prevotella population has been seen to increase and remain stable post-RYGB at a quantity seen in lean control subjects. The higher the increase in the proportions of Bacteroides/Prevotella, the greater the reduction in body fat mass and plasma leptin [44].

Performing a RYGB in a rodent model markedly alters the composition of the distal gut microbiota as early as one week after surgery, a change that progressed over time and stabilised after five weeks independent of diet [45]. Specific changes in the gut microbial community were demonstrated including enrichment in three distinct taxonomic groups; evident at phylum level—Bacteroidetes, Verrucomicrobia, and Proteobacteria; to genus level—*Alistipes*, *Akkermansia*, and *Escherichia* [45]. A substantial increase in the abundance of Proteobacteria specifically the species *Enterobacter hormaechei* and a reduction in Firmicutes and Bacteroidetes has also been observed [46].

Liou et al. [45], performed FMT procedures using microbiota extracted from rodents who had a RYGB procedure. The control arm facilitated mice that had received a sham RYGB. It was demonstrated that the rodent cohort who had received the treatment-FMT displayed substantial weight reduction and loss of fat mass compared to their counterparts receiving sham-FMT [45]. Further research focusing on GF-rodents colonised with RYGB microbiota illustrates the accumulation of 43% less body fat and a lower respiratory quotient than rodents colonised with ‘obese’ microbiota. This particular finding suggests the reduction in usage of carbohydrates and increased utilisation of lipids as a potential energy source [43].. In both studies, a decrease in adiposity and body weight without a change in chow intake was observed suggesting RYGB-associated microbiota may either reduce the ability to harvest energy from the diet or produce signals regulating energy expenditure and lipid metabolism [45].

### Future manipulation of microbiota

As the evolving exploration for causality between obesity and microbiota continues, attention has been diverted to the search for techniques in microbiota manipulation with the objective of restoring a balanced gut microbiota community. FMT techniques have been refined and involve the transfer of carefully screened faecal material containing microbiota from a healthy donor into an identified ‘diseased’ patient with the intention of cure [127]. Probiotics and prebiotics are also proposed methods to manipulate the gut microbiota population in order to improve metabolic conditions, however FMT is considered to have the potential for being more successful. FMT has the ability to transfer entire donor microbiota communities, including their metabolites, to the identified recipient, with the perceived enhanced capability to correct microbiota disruption over single microbial targets such as probiotic supplementation [128].

FMT studies aiming to improve metabolic parameters are increasing in number. Vrieze et al., were the first group (2012) to perform human FMT using treatment-naive subjects diagnosed with metabolic syndrome [47]. Eighteen subjects were randomised to receive either FMT produced from lean male donors (BMI < 23) or autologous transfusions. Subjects who received allogenic transfusions were noted to have improved peripheral insulin sensitivity (after 6 weeks) but this effect deteriorated with time and there was considerable individual variability. Allogenic recipients demonstrated higher abundances of butyrate-producing bacteria (the species *Roseburia intestinalis*) post-treatment [47].

Two randomised studies investigated the effects of using lean allogenic FMT in subjects diagnosed with metabolic syndrome [129, 130]. Both studies failed to show any improvement in metabolic parameters or subject physiology. However, it was noted that recipients of the lean allogenic FMT mostly, but not all were found to have gut microbiota composition that shifted towards an appearance similar to the donor’s profile implying unsuccessful engraftment [129, 130] (see Table 2).

**Table 2:** Faecal Microbiota Transplantation usage in human studies of obesity/metabolic syndrome

Paper	No. patient	Demographic	Type of study	Mode of delivery	Country of Study	Frozen / fresh	Outcomes	Significant Adverse Events
Allegretti et al. [129]	22 11 = allogenic lean FMT 11 = placebo	Obese BMI > 35 without metabolic complications	DBRCT* 1:1	Capsules	USA	Unrelated donor Frozen stool	No change in GLP1 in either group No significant changes in obesity biomarkers No changes in BMI Sustained shift of microbiome towards donor profile	Nil
Vrieze et al. [47]	18 9 = Lean allogenic 9 = autologous FMT	Treatment naïve males with metabolic syndrome	RCT* placebo controlled pilot study 1:1	Nasoduodenal tube	Netherlands	Unrelated donor Fresh stool	Improvement in peripheral insulin sensitivity at 6 weeks in allogenic FMT Gut bacterial diversity significantly increased post allogenic FMT	Nil
Smits et al. [130]	20 10 = allogenic lean vegan FMT 10 = autologous FMT	Male cohort with metabolic syndrome	DBRCT pilot study 1:1	Nasoduodenal tube	Netherlands	Unrelated donor Fresh stool	No changes in faecal diversity 2 weeks post FMT Allogenic FMT shifted microbiome profile towards vegan No changes in vascular inflammation	Nil

**DBRCT: double-blinded randomised control trial, RCT: randomised control trial**

It has been estimated that a sample size of 1700 subjects per study is likely needed to adequately assess the relationship between obesity and microbiota composition after correcting for variables (17). Of the research that has already been completed, carefully controlled FMT has satisfied the safety requirements but with underwhelming clinical findings at present. FMT has demonstrated its potential for restoring both gut-microbiota composition and functionality however better understanding of the mechanisms through which these alterations translate into metabolic outcomes is still unknown. With the continued introduction of advancing technology and an ever-increasing co-morbid population, further exploration is still required for the clarification of gut microbiota causality before the routine establishment of microbiota manipulation occurs.

### Study strengths and limitations

This review updates previous literature by bringing together the central microbiomic theories underpinning obesity from a metabolome perspective. It is limited by a persistent lack of consensus understanding of the mechanistics by which the microbiota exert their obesogenic effect, and given the heterogeneity of the literature was conducted in a non-systematic way.

## Conclusion

The gut microbiota has enormous metabolic capacity behaving as a central modulator in the contribution towards obesity. Research clearly indicates significant discrepancies in determining the cause or effect relationship between the gut microbiota and obesity. The relationship has partly been established at structural level, however it seems that functionality rather than the composition of microbiota populations may contain the answers to the mechanisms underlying obesity. This review has discussed central mechanisms involving the gut microbiota in their ability to promote obesity development such as the inhibition of Fiaf, altered production of SCFAs, heightened inflammatory pathways, increased gut permeability with resulting endotoxaemia, and disrupted BA metabolism as future druggable and modifiable targets. Given the increasing output of controlled research we should soon have a better understanding of the gut microbiota:obesity association and whether clinically modulating the gut microbiome through FMT will provide a new therapeutic option for the management of this complex disorder.

## Data Availability

Data sharing not applicable to this article as no datasets were generated or analysed during the completion of this review.

## Abbreviations

AKT: protein kinase B
ATP: adenosine triphosphate
BA: bile acid
BMI: body mass index
BSH: bile salt hydrolases
CA: cholic acid
cAMP: cyclic adenosine monophosphate
CB1: cannabinoid receptor type-1
CD14: cluster of differentiation 14
CDCA: chenodeoxycholic acid
CONV-D: conventionalised GF-mice
DCA: deoxycholic acid
DIO2: type 2 deiodinase
Fiaf: fasting-induced adipose factor
FGF-19: fibroblast growth factor 19
FXR: farnesoid X receptor
FMT: faecal microbiota transplant
GF: germ free
GIP: glucose dependent insulinotropic polypeptide
GIT: gastrointestinal tract
GLP-1: glucagon-like peptide 1
GPR: G protein coupled receptor
H_2_: hydrogen
IGN: intestinal gluconeogenesis
IL: interleukin
iNOS: inducible nitric oxide synthase
Kg: kilograms
LBL: lipoprotein lipase
LCA: lithocholic acid
LGC: low gene count
LPS: lipopolysaccharide
NF- κB: nuclear factor kappa-light-chain-enhancer of activated B cells
NPY: neuropeptide Y
RYGB: Roux-en-Y gastric bypass
PGC-1: peroxisome proliferator-activated receptor gamma coactivator one
PPARα: peroxisome proliferator-activated receptor alpha
PYY: peptide tyrosine-tyrosine
SCFA: short chain fatty acid
TGR5: G protein-coupled receptor
TNF: tumour necrosis factor
UDCA: ursodeoxycholic acid
VLDL: very low density lipoprotein
WAT: white adipose tissue
